# Knockdown of the Ribosomal Protein eL29 in Mammalian Cells Leads to Significant Changes in Gene Expression at the Transcription Level

**DOI:** 10.3390/cells9051228

**Published:** 2020-05-15

**Authors:** Alexander V. Gopanenko, Alena V. Kolobova, Maria I. Meschaninova, Alya G. Venyaminova, Alexey E. Tupikin, Marsel R. Kabilov, Alexey A. Malygin, Galina G. Karpova

**Affiliations:** 1 Institute of Chemical Biology and Fundamental Medicine, Siberian Branch of the Russian Academy of Sciences, Prospekt Lavrentieva 8, Novosibirsk 630090, Russia; alexandr.gopanenko@yandex.ru (A.V.G.); alyona_kolobova@rambler.ru (A.V.K.); mesch@niboch.nsc.ru (M.I.M.); ven@niboch.nsc.ru (A.G.V.); alenare@niboch.nsc.ru (A.E.T.); kabilov@niboch.nsc.ru (M.R.K.); malygin@niboch.nsc.ru (A.A.M.); 2 Department of Molecular Biology, Novosibirsk State University, Novosibirsk 630090, Russia

**Keywords:** HEK293 cells, knockdown of ribosomal protein eL29, next-generation sequencing, RNA-seq, differential gene expression, eL29-dependent genes, eL29-related processes

## Abstract

An imbalance in the synthesis of ribosomal proteins can lead to the disruption of various cellular processes. For mammalian cells, it has been shown that the level of the eukaryote-specific ribosomal protein eL29, also known as the one interacting with heparin/heparan sulfate, substantially affects their growth. Moreover, in animals lacking this protein, a number of anatomical abnormalities have been observed. Here, we applied next-generation RNA sequencing to HEK293 cells transfected with siRNAs specific for the mRNA of eL29 to determine what changes occur in the transcriptome profile with a decrease in the level of the target protein. We showed that an approximately 2.5-fold decrease in the content of eL29 leads to statistically significant changes in the expression of more than a thousand genes at the transcription level, without a noticeable effect on cell viability, rRNA level, and global translation. The set of eL29-dependent genes included both up-regulated and down-regulated ones, among which there are those previously identified as targets for proteins implicated in oncogenesis. Thus, our findings demonstrate that an insufficiency of eL29 in mammalian cells causes a significant reorganization of gene expression, thereby highlighting the relationship between the cellular balance of eL29 and the activities of certain genes.

## 1. Introduction

Ribosomal proteins play a pivotal role in maintaining the architecture of large (60S) and small (40S) ribosomal subunits and are involved in the operation of the translational machinery, being its constituents [1,2,3,4,5]. A typical mammalian ribosome contains 80 individual ribosomal proteins [5], most of which are essential for cell viability. The deficiency of ribosomal proteins in cells usually causes significant disturbances in the processing of rRNA and the assembly of ribosomal subunits, which leads to a reduction in the population of ribosomes and, as a consequence, to a decrease in the rate of protein synthesis [6,7,8]. Meanwhile, there are ribosomal proteins whose deficiency in cells does not significantly affect the level of ribosomal subunits, which allows these proteins to be classified as non-essential ones [6,9,10,11,12,13,14]. Such ribosomal proteins are eS25, RACK1, uL11, eL29, eL41 and some others. There are data indicating that these proteins are important players in a number of cellular processes unrelated to their participation in translation as components of ribosomes [15,16,17]. For example, in NIH 3T3 cells, RACK1 colocalizes with proto-oncogene tyrosine-protein kinase Src (c-Src) at the plasma membrane and functions as a substrate and inhibitor of c-Src [17]. In *Caenorhabditis elegans*, uL11 controls its own synthesis by inhibiting the splicing of its pre-mRNA [15]. To date, the most impressive are the data obtained for the mammalian ribosomal protein eL29, which has no homologues in eubacteria and archaea [18].

The eL29 protein, a component of the 60S ribosomal subunit, is also known as one interacting with heparin/heparan sulfate, found earlier on the surface of uterine endothelial cells and later on the surfaces of other types of epithelial cells [19,20,21]. It has been shown that eL29, when heparin/heparan-bound, is involved in a variety of cellular processes, such as cell-to-cell communication, adhesion, differentiation, proliferation and blood coagulation modulation [22,23,24,25]. In endothelial cells lacking αvβ3 integrin, vascular sprouting has been found to be inhibited with a deficiency or loss of eL29 [26]. It has turned out that in animals the absence of eL29 leads to the appearance of phenotypes with various anatomical anomalies [27,28]. In particular, it has been demonstrated that mice with the homozygous eL29-null mutation had less body weight and proportionally reduced sizes of organs and skeleton, along with the increased fragility of bones and teeth and other defects, compared to normal mice of the same age [27,28,29,30]. The authors of these studies have suggested that all observed abnormalities are associated with ribosome insufficiency caused by the lack of eL29 in the mice. Alternatively, disturbances in the cellular processes related to the regulation of gene expression, which could have occurred due to the absence of eL29, have led to the appearance of the aforementioned defects [27,28,29,30].

An appropriate method for determining genes whose expression could depend on the content of ribosomal proteins in the cells is next-generation sequencing (NGS) of RNA at the level of whole transcriptome (RNA-seq) (see, e.g., [31,32,33]). In this study, using the RNA-seq method, we revealed changes in the transcriptome profile of HEK293 cells, which occurred due to a decrease in the level of ribosomal protein eL29 through RNA interference triggered by the transfection of the cells with siRNAs specific for its mRNA. We found that eL29 deficiency does not considerably affect cell viability, rRNA content and global translation, but it leads to significant changes in the expression of a large number of genes at the transcription level. The expression of one gene group was enhanced when the cellular content of eL29 decreased, while the expression levels of the genes from the another group were reduced, which allowed classifying the genes of these groups as up- and down-regulated ones, respectively. Among the eL29-dependent genes, there were also those known as targets for proteins related to oncogenesis. The results prove that the deficiency of the ribosomal protein eL29 in mammalian cells leads to significant changes in gene expression, exposing genes whose activities depend on the content of eL29.

## 2. Materials and Methods

### 2.1. Preparation of siRNAs

The list of oligoribonucleotides used as siRNAs is presented in Supplementary Table S1. These oligomers were obtained using RNA phosphoramidites (Glen Research, Sterling, VA, USA) and the synthesis protocol for the automatic DNA/RNA synthesizer ASM-800 (Bioset, Novosibirsk, Russia). After purification by 20% polyacrylamide gel electrophoresis (PAGE) in the presence of 8 M urea, they were desalted on a Step-Pac C18 cartridge (Millipore, Temecula, CA, USA) and isolated as lithium salts.

### 2.2. Cell Culture and eL29 Knockdown

HEK293 cells were cultured in DMEM (Invitrogen) supplemented with 10% FBS (Gibco) in a 5%-CO_2_ incubator at 37 °C. When the confluency reached approximately 60%, the cells were transfected with siRNAs specific for the mRNA of eL29 using Lipofectamine 3000 (Thermo Fisher Scientific) according to the manufacturer’s protocol. In a control experiment, the cells were transfected with scrambled siRNA. Two days after the transfection, the culture medium was aspirated, and the cells were washed with ice-cold PBS and then collected by centrifugation at 500× *g* for 5 min at 4 °C. To confirm the knockdown of eL29, its content in the cells was determined by western blotting using specific rabbit polyclonal antibodies against eL29 (#PA5-27545) (Thermo Fisher Scientific). Briefly, the cell pellet (see above) was lysed in 20 mM Tris-HCl (pH 7.5) buffer containing 150 mM NaCl, 1% NP40 and 5% glycerol and incubated for 10 min at 0 °C. The cell lysate was then clarified by centrifugation at 20,000 g at 4 °C for 10 min and the resulting supernatant was supplemented with Laemmli sample buffer, followed by incubation for 10 min at 80 °C. The proteins were resolved by 12–15% PAGE in the presence of sodium dodecyl sulfate (SDS) and transferred onto a nitrocellulose membrane (0.45 μm). The blot was sequentially probed with primary and corresponding peroxidase-conjugated secondary antibodies and developed with an ECL Western Blotting Substrate Kit (Thermo Fisher Scientific) followed by exposure to an X-ray film or the ChemiDoc XRS system (Bio-Rad).

### 2.3. Determination of eL29 and rRNA Contents in eL29-Knocked Down Cells

HEK293 cells grown in a 12-well plate were transfected, washed with ice-cold PBS and collected by centrifugation as described above. Total RNA and protein were isolated by treating the cell pellets with 200 μl of TRIzol reagent (Thermo Fisher Scientific) according to the manufacturer’s protocol. The RNA samples were resolved by 1% agarose gel electrophoresis, followed by staining with ethidium bromide, and visualized using the ChemiDoc XRS system (Bio-Rad). The total protein was analyzed for eL29 content by western blotting as described above.

### 2.4. Estimation of the Effect of eL29 Knockdown on Cellular Surveillance and Proliferation

To determine the effect of eL29 knockdown in HEK293 cells on their phenotype, the 3-(4,5-dimethylthiazol-2-yl)-2,5-diphenyl tetrazolium bromide (MTT) test was performed using the EZ4U cell proliferation assay (Biomedica) according to the manufacturer’s protocol. The microplate reader Polarstar Optima (BMG Labtech) was exploited to detect the cell fluorescence. HEK293 cells grown in a 96-well plate and transfected as described above were used for the MTT test, which was carried out with cell samples taken immediately after transfection, as well as 1 and 2 days after it.

### 2.5. Analysis of the Content of Ribosomal Proteins in the Lysate and Polysome Profile Fractions of Knockdown Cells

Polysome profiles were obtained as described in [34], with minor modifications. For a typical experiment, the HEK293 cell lysate was centrifuged in a 7–47% sucrose gradient at 100,000 g for 4 h at 4 °C using a Beckman SW40 rotor and fractionated with measuring UV absorbance at 260 nm. Total protein from fractions corresponding to 80S ribosomes or polysomes was isolated using StrataClean granules (Stratagene), resolved by SDS-PAGE and analyzed by western blotting using the aforementioned anti-eL29 antibodies, as well as mouse polyclonal antibodies against human uS2 (kindly gifted by Dr. I. Shatsky) and goat antiserum against rat ribosomal protein uL18 (kindly gifted by Dr. J. Stahl) as described above. Analysis of the content of ribosomal proteins in the cell lysate was performed by western blotting with the use of rabbit polyclonal antibodies against ribosomal protein uL5 from Nordic BioSite (#16277-1-AP) and home-made rabbit polyclonal antisera against human ribosomal proteins uS15 and eS10. Antibodies against GAPDH were from Proteintech (#60004-1-Ig). Images of the blots were acquired using the VersaDoc Imaging System (Bio-Rad) and analyzed using the Quantity one software version 4.6.3 (Bio-Rad). Calculations of the standard error of the mean (CEM) and Student’s t-test were performed using GraphPad Prism version 8.3.0 (GraphPad Sofware).

### 2.6. RNA-Seq Analysis

RNA-seq was performed in SB RAS Genomics Core Facility (ICBFM SB RAS, Novosibirsk). For a typical experiment carried out in three biological replicates, HEK293 cells in two 10 cm Petri dishes were used, one of which contained the cells transfected with siRNAs specific for the mRNA of eL29, and the other was with the cells transfected with scrambled siRNA. Two days after transfection, the culture medium was supplemented with harringtonine to a final concentration of 2 μg/mL for 2 min, and then cycloheximide was added to a final concentration of 100 μg/mL for 1 min. After this, the culture medium was removed and the cells were briefly washed with ice-cold PBS and collected by centrifugation at 500× *g* at 4 °C for 5 min. The cell pellet was lysed in TRIzol (Invitrogen) with subsequent isolation of total RNA by Purelink RNA Micro Scale Kit (Invitrogen) and DNase treatment (DNASE70, Sigma). RNA integrity index (RIN) was assessed using the RNA 6000 Pico Kit and Bioanalyzer 2100 (Agilent), and RNA quantification was carried out using NanoDrop 1000 (Thermo Scientific) and Qubit (Invitrogen). For the 1 µg of each isolated RNA sample, polyA enrichment was performed by the NEBNext Poly(A) mRNA Magnetic Isolation Module (NEB), followed by the preparation of cDNA libraries using the NEBNext Ultra II Directional RNA Library Prep Kit for Illumina (NEB). After quality control on a Bioanalyzer 2100 (High Sensitivity DNA Kit, Agilent), the DNA libraries were quantified by qPCR (CFX96 Touch, Bio-Rad), pooled in equimolar amounts and sequenced on HiSeq 2500 (Illumina) using 2 × 100 bp chemistry. The basic characteristics of the cDNA libraries are presented in Supplementary Table S2. The RNA-seq read data reported in this study were submitted to the GenBank under the study accession PRJNA611889.

### 2.7. Validation of RNA-Seq Data by Reverse Transcription-qPCR

Reverse transcription (RT) was carried out using 5 μg of total RNA extracted from the respective cells as described above, 100 pmol of random hexamer primer and 100 U of Maxima H Minus reverse transcriptase (Thermo Fisher Scientific) according to the manufacturer’s protocol. PCR with the obtained cDNA was performed on LightCycler 96 (Roche) using SYTO9 fluorescent dye (Thermo Fisher Scientific), HS-Taq polymerase (Biolabmix) and gene-specific primers (Supplementary Table S3). The experiments were carried out in three biological replicates. Relative quantification of expression levels of genes of interest was determined by normalizing them to that of the *GAPDH* gene using the LightCycler 96 integrated software. The data obtained were analyzed by GraphPad Prism version 8.3.0 using the Mann–Whiney test. Pearson’s correlation between the Log2FoldChage (LFC) values of the tested gene set calculated in Excel from the RT-qPCR data and the LFC values determined for the same genes from the RNA-seq data analysis (see below) was estimated using the *cor.test* function from the R Stats package.

### 2.8. Bioinformatic Analysis

The demultiplexed fastq files were quality-checked using the FastQC tool (v. 0.11.9), and a summary of quality metrics was generated using the MultiQC tool (v.1.8). Fastq reads were analyzed using tools of the CLC GW 12.0 software (Qiagen). Reads were filtered by both the quality (by default) and adapter sequences, and then mapped to the human reference genome (hg38) with Ensembl annotation GRCh38.93 by the RNA-seq analysis tool (Strand specific = Reverse and other parameters by default). The generated BAM files were used for subsequent analysis steps. The pattern of distribution of reads across genomic features was obtained using the systemPipeR package (v 1.20.0). Read counting was performed using the featureCounts function (isGTFAnnotationFile = TRUE, isPairedEnd = TRUE, strandSpecific = 2 (reversely stranded)) from the Rsubread package (v.1.22.2).

The resulting raw counts were used for differential gene expression analysis using the DESeq2 package (v. 1.26.0). The design formula was ~ batch + condition, where the batch is the day of the experiment (I for replicates 1 and 2 that were made in one day, and II for replicate 3 that was made in another day), and the conditions were "norm" vs "eL29 knockdown" (where "norm" corresponds to the transfection of HEK293 cells with scrambled siRNA). The “batch” was added for the subsequent removal of any batch effects (see [35]), which could occur due to different replicate preparation times, using the removeBatchEffect function from the Limma package (v. 3.42.0). The adjusted p-value (p.adj) parameters were obtained after the correction of the p-value for multiple testing using the Benjamini and Hochberg method, which is implemented in DESeq2 by default. The plot with the data of the principle component analysis (PCA) of gene expression was made using the plotPCA function with default values (ntop = 500), as described in the DESeq2 vignette. Euclidean distances between the analyzed samples (replicates) were calculated using the dist function of R. A heatmap of hierarchical clustering based on the calculated Euclidean distances was generated using the pheatmap function from the Pheatmap package (v. 1.0.12). Differences in gene expression levels were considered as statistically significant when p.adj values of the genes were less than 0.01, and the absolute values of their LFCs calculated by DESeq2 were more than 0.322 (i.e., gene expression levels were changed by more than 25%). To identify differently expressed genes with statistically significant changes in their levels (DEGs), the gene set generated by DESeq2 was filtered by the above p.adj values and LFC ones more than 0.322 and less than −0.322. These LFC values corresponded to up-regulated and down-regulated genes, for which, respectively, an increase or decrease in their expression levels in eL29-reduced cells was more than 25% of those in cells transfected with scrambled siRNA. The MA plot with differential gene expression analysis data was created using the plotMA function from the DESeq2 package with the alpha parameter = 0.01.

Pathway and Gene Ontology (GO) enrichment analyses were made with the utilization of sets of up-regulated and down-regulated DEGs. Pathway analysis was performed using the *enrichPathway* function (pvalueCutoff = 0.1) from the ReactomePA package (v.1.30.0). GO enrichment analysis was conducted using the GeneOntology web service [36] (the analysis type: PANTHER Overrepresentation Test (Released 20190711, Annotation Version and Release Date: GO Ontology database Released 2019-12-09, Reference List: Homo sapiens (all genes in the database)), Test Type: FISHER, Correction: FDR).

To analyze the overlap of DEG sets with those of p53 and c-Myc target genes, the previously described sets of 343 and 1469 target genes for p53 and c-Myc, respectively [37,38], were used. Other manipulations with the data obtained were performed using custom R scripts with default R functions. 

## 3. Results

### 3.1. Knockdown of the Ribosomal Protein eL29 in HEK293 Cells

**Figure 1:**
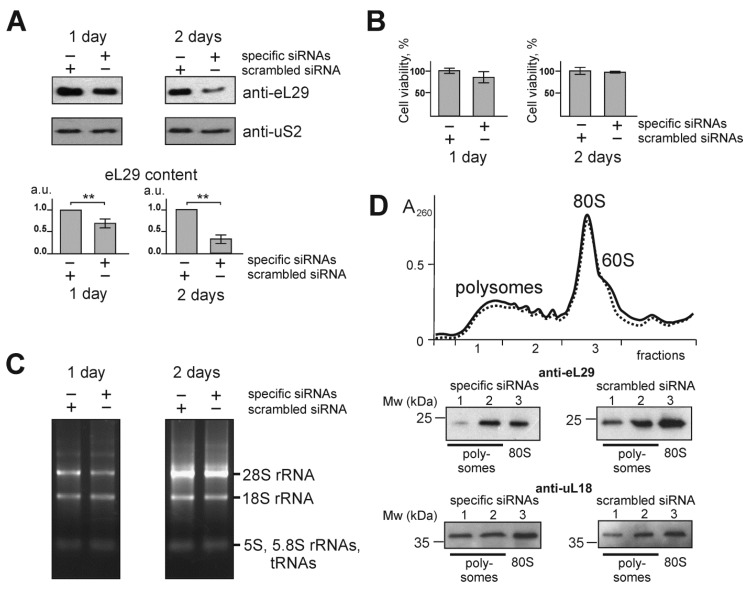
Characterization of HEK293 cells knocked down of ribosomal protein eL29. (**A**) Western blot analysis of the levels of ribosomal proteins eL29 and uS2 (as a reference) in cells transfected with siRNAs specific for eL29 mRNA with scrambled control, 1 and 2 days after transfection. The diagrams show the western blot data in triplicate as the mean of arbitrary units (a.u.) ± CEM (** *p* < 0.01, calculated using Student’s t-test). (**B**) Histograms of MTT assay of cells transfected with scrambled or eL29 mRNA-specific siRNAs. (**C**) Electrophoretic analysis of total RNA samples extracted from cells transfected with the above siRNAs on a 0.5% agarose gel stained with ethidium bromide. (**D**) Polysome profiles obtained by centrifugation of lysates of cells transfected with scrambled (solid line) and eL29 mRNA-specific (dashed line) siRNAs in sucrose density gradient with marked peaks corresponding to 60S subunits, 80S ribosomes and polysomes. Western-blot analysis of gradient fractions for the presence of eL29 and uL18 (as a reference). Lanes 1, 2 and 3 correspond to the sucrose gradient fractions containing “heavy” polysomes (>5 ribosomes per mRNA), “light” polysomes (≤5 ribosomes per mRNA) and 80S ribosomes (together with a small portion of 60S subunits), respectively.

To reduce the level of ribosomal protein eL29 in HEK293 cells, the RNAi approach was used. To this end, the cells at approximately 60% confluence were transfected with siRNAs targeted to the mRNA of this protein, while scrambled siRNA was utilized in a control experiment (see Supplementary Table S1). Time-course changes in the level of eL29 in the transfected cells were determined by western blotting using the respective specific antibodies, and those in cell viability were assessed using the MTT test. One and two days after transfection, the level of eL29 decreased by about 1.5 and 2.5 times, respectively, compared to that in the cells transfected with scrambled siRNA, and the level of the ribosomal protein uS2, examined as a reference, remained virtually unchanged (Figure 1A). The MTT test did not reveal significant differences in the viability of HEK293 cells transfected with scrambled and eL29 mRNA-specific siRNAs (Figure 1B), and, in addition, no differences were found between the total rRNA samples isolated from these cells (Figure 1C). Finally, the general shape of the sucrose gradient polysome profile from the eL29-reduced cells was almost identical to that for the cells with a normal level of eL29, although levels of this protein were reduced in the respective pooled gradient fractions corresponding to ribosomes or polysomes (Figure 1D). All this implied that eL29-null ribosomes were active in translation. Thus, one can conclude that HEK293 cells knocked down for eL29 are an appropriate model to study the effect of a decrease in the level of eL29 on a mammalian cell transcriptome.

### 3.2. RNA-Seq Analysis of eL29-Knocked Down HEK293 Cells

For an in-depth analysis of the effect of eL29 knockdown on the transcriptome, HEK293 cells were transfected with the respective siRNAs in three biological replicates, with scrambled control for each one, for 2 days, followed by RNA extraction using TRIzol reagent. NGS of cDNA libraries derived from the resulting RNA samples was performed on the HiSeq 2500 platform using 100 bp paired-end reads (Supplementary Table S2). Analysis of the raw RNA-seq data using the FastQC tool confirmed the high quality of the sequencing reads (Q-score for all samples was >32) (Supplementary Figure S1A,B ). These reads were mapped to the reference human genome using CLC GW, and downstream analysis was conducted using Bioconductor packages and custom R-scripts. The distribution of the mapped reads across the most abundant protein-coding transcripts is presented in Supplementary Figure S1C. It was found that the reads predominantly fell to genes, rather than intergenic regions (Supplementary Figure S1D).

**Figure 2:**
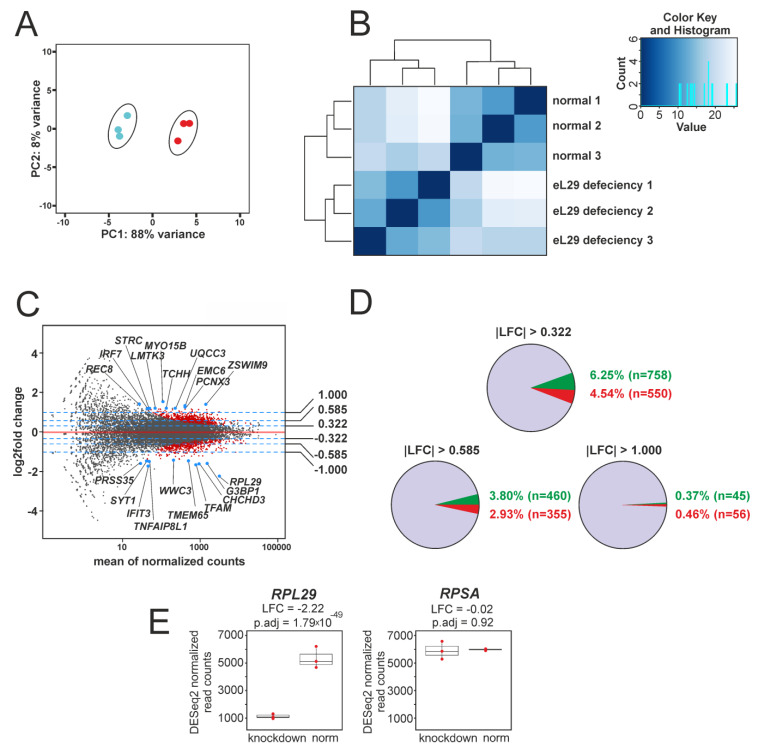
Summary of the results of exploratory RNA-seq data analysis. (**A**) PCA plot of gene expression data obtained via RNA-seq data for three biological replicates corresponding to the samples from HEK293 cells transfected with scrambled siRNA (blue dots) and from those knocked down of eL29 (red dots). (**B**) A heatmap based on the Euclidean distance matrix between the replicates of the above samples, designated as “normal” and “eL29 deficiency”, respectively, displaying hierarchical gene clustering for each replicate. The color key and the histogram panel show the total number of RNA-seq datasets as a function of their Euclidean distance values. (**C**) MA plot of the LFCs values of all genes, exposing the results of differential gene expression analysis with RNA-seq data from the above replicates. Normalized mean read counts for the genes are plotted along the x-axis, and gene LFCs values are plotted along the y-axis. Red dots correspond to genes with statistically significant changes in expression levels (p.adj values <0.01 and LFCs absolute values >0.322) (called as DEGs, see Section 2.8); all other genes are marked by black dots. The top 20 DEGs (10 up- and 10 down-regulated ones in accordance with their LFCs) are shown as blue dots with labels. The blue dashed lines indicate the threshold LFCs values: 1, 0.585, 0.322, −0.322, −0.585 and −1, which correspond to 100%, 50% and 25% changes in gene expression. (**D**) A summary of DESeq2 analysis results. The diagrams show the contents of up-regulated and down-regulated DEGs displayed by green and red slices, respectively, as a percentage of the total number of analyzed genes, in accordance with their LFCs values. (**E**) A comparative representation of the changes in the expression of the *RPL29* and *RPSA* genes (encoding the ribosomal proteins L29 and uS2, respectively), found in the DESeq2 analysis and shown as boxplots. Red dots display the values of normalized read counts for each sample.

Examination of the RNA-seq data using PCA and distance matrix analysis showed a high quality of clustering between biological replicates (Figure 2A,B), implying that the data obtained are suitable for further application. In this line, differential gene expression analysis was performed on a set of more than 12,000 genes generated using DESeq2 (Supplementary Table S4). The resulting gene set contained 1308 protein-coding ones (Supplementary Table S5), designated as DEGs in the Materials and Methods section. An MA plot illustrating the data of differential gene expression analysis is presented in Figure 2C. The shares of up-regulated and down-regulated DEGs, the expression of which in eL29-knocked down cells is accordingly enhanced or weakened compared to that in cells transfected with scrambled siRNA, are shown in Figure 2D. DESeq2 analysis data displaying changes in the expression of genes encoding the ribosomal proteins eL29 and uS2 (examined as a reference in Figure 1A) in eL29-reduced cells, compared with their expression in cells transfected with scrambled siRNA, are presented in Figure 2E. These data, together with those from Figure 1A, imply that in eL29-knocked down HEK293 cells, the level of eL29 mRNA is significantly lower than that of eL29 itself. The discrepancy between the levels of eL29 and its mRNA, observed two days after transfection, indicates that the synthesis rate of eL29 exceeds that of its degradation. For several DEGs with high baseMean values, namely *CHCHD3, CLIC4, G3BP1* and *TFAM*, whose expression levels were reduced, and the *RAD23A* gene, whose expression was enhanced, RNA-seq data were validated by RT-qPCR (Supplementary Figure S2). A high correlation was found between the values of changes in the expression levels of the above genes, estimated from RNA-seq and RT-qPCR data (Pearson’s correlation (r) = 0.913; *p*-value = 0.004).

It should be noted that among DEGs, besides *RPL29*, genes of several other ribosomal proteins were detected (Supplementary Table S6 and Figure S3A). These are the up-regulated genes *RPL23A*, *RPS5* and *RPS21* encoding proteins uL23 (L23A), uS7 (S5) and eS21 (S21), as well as the down-regulated ones *RPL34*, *RPL5, RPL11*, *RPL12* and *RPL22* encoding proteins eL34 (L34), uL18 (L5), uL5 (L11), uL11 (L12) and eL22 (L22), respectively. For one of these proteins, it was shown that its level in eL29-deficient HEK293 cells was indeed altered. In particular, the level of uL5, whose gene turned out down-regulated, was reduced (Supplementary Figure S3B) to an extent comparable to that of the decrease in the level of expression of its coding gene (Supplementary Figure S3A).

### 3.3. Cellular Processes Associated with eL29-Dependent DEGs

**Figure 3:**
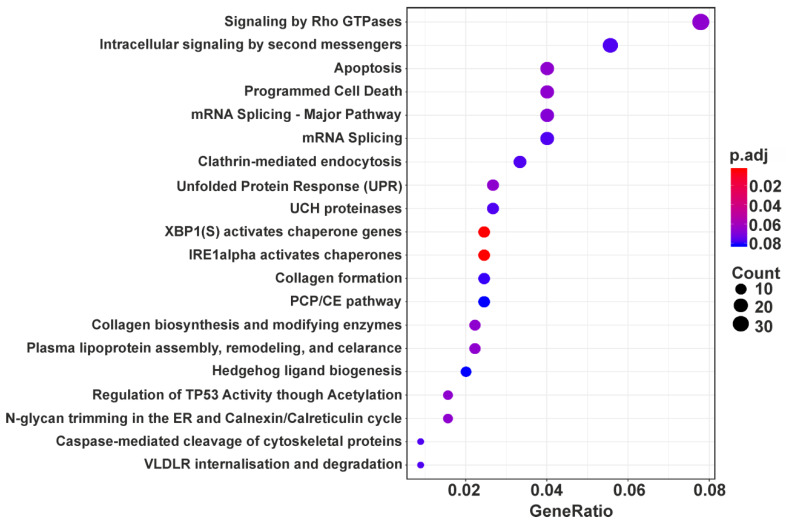
Dotplot enrichment map showing cellular pathways associated with up-regulated DEGs. The top 20 by DEG counts are presented from 38 pathways with p.adj values <0.1 found for the above DEGs (Supplementary Table S7). The color of the dot depends on the value of p.adj, and its size is determined by the number of DEGs related to the respective pathway in the analyzed set of DEGs (map color keys along with dot size ones are shown on the right).

A total of 758 up-regulated and 550 down-regulated DEGs were identified and GO enrichment and ReactomePA pathway analyses were applied to all the DEGs to determine the biological processes and pathways in which they are involved. The first one performed on the set of up-regulated DEGs revealed five processes in which these DEGs are enriched. These are protein quality control for misfolded or incompletely synthesized proteins (*UFD1*, *RNF5*, *HDAC6*, etc.), response to endoplasmic reticulum stress (*CUL7*, *NFE2L1*, *SRPRA*, etc.), mitochondrion organization (*COX17*, *LETM1*, *UQCC3*, etc.), regulation of GTPase activity (*AKT2*, *GPSM1*, *GDI1*, etc.) and regulation of mitotic cell cycle (*CDK10*, *INCENP*, *DCTN1*, etc.) (Table 1). Pathway analysis applied to the same set of DEGs displayed a wider range of DEG-related processes, including intracellular signal transmission, apoptosis, splicing, collagen formation, and some others, among which are also those determined using GO enrichment analysis (Figure 3 and Supplementary Table S7).

**Figure 4:**
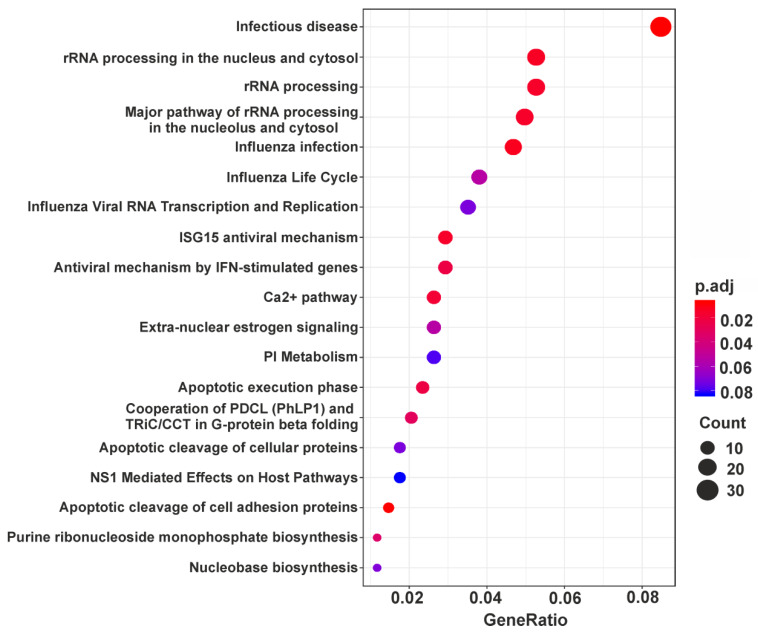
Dotplot enrichment map representing cellular pathways associated with down-regulated DEGs. All 19 pathways with p.adj values <0.1 found for the above DEGs (Supplementary Table S7) are shown. Designations are the same as in Figure 3.

Unlike up-regulated DEGs, down-regulated ones turned out to be enriched in more than a dozen processes. It is noteworthy that among them there are processes responsible for making ribosomes, namely, RNA (including rRNA) processing and ribonucleoprotein complexes (including ribosome) biogenesis (*WDR12*, *DDX21*, *NPM1*, etc.), as well as those related to the functioning of a protein-synthesizing machinery, such as translation initiation, along with the regulation of translation (*EIF4EBP1*, *PTBP3*, *EIF2AK2*, etc.) (Table 1). Besides, GO enrichment analysis showed that down-regulated DEGs can be implicated in nucleobase-containing small molecule metabolic process (*PPAT, NADK2, NAMPT,* etc.), nuclear transport (*NUP37, NUP58, XPOT,* etc.), organophosphate (*OCRL, PLS1, PIGX,* etc.) and phospholipid biosynthetic processes and actin filament-based process (*PDLIM5, SHROOM3, MYO1D,* etc.) (Table 1). Finally, as can be seen in the table, among the processes defined as associated with down-regulated DEGs, there are those, such as positive regulation of cell projection organization (*MNS1, WASL, OCLN,* etc.), protein localization to membrane (*LIN7C, VAMP7, CD24,* etc.), viral process (*MICB, AP1S2, CR2,* etc.), as well as regulation of cellular catabolic process (*NAMPT, PGAM1, MET,* etc.) and regulation of cellular response to stress (*CD44, HIF1A, UBE2N,* etc.). In addition, pathway analysis revealed the involvement of down-regulated DEGs in cellular response to infections, Ca^2+^ pathway, estrogen signaling, as well as a number of others, including some of those found using GO enrichment analysis (Figure 4 and Supplementary Table S7).

All this suggests that with a deficiency of eL29, the cell undergoes a substantial reorganization that allows it to adjust internal processes in order to overcome the negative consequences caused by the imbalance of this protein. For example, one can see that the cell is trying to reduce the activity of the genes responsible for the synthesis of ribosomes and translation factors, which may certainly lead to a decrease in the translation rate and, accordingly, limit the cell growth. On the other hand, the stimulation of genes related to signaling, activation of chaperones, mitochondrion organization and collagen formation, which occurs with a decrease in the level of eL29, indicates that the cell is attempting to correct the disorders associated with the organization of membranes and the cytoskeleton.

### 3.4. DEGs as Targets for p53 and c-Myc

Since the disruption of ribosome biogenesis due to an imbalance of ribosomal proteins usually leads to the activation of the p53 tumor suppressor by sequestering the ubiquitin ligase MDM2 or several other ways [39,40], it was reasonable to expect changes in expression levels of *TP53* or *MDM2* genes upon eL29 knockdown in HEK293 cells. As can be seen in Supplementary Table S4, none of these genes is contained in the set of DEGs revealed for eL29-reduced cells, but instead, there are genes previously assigned to p53 targets [37]. These are 23 up-regulated DEGs (*BAX, CES2, XPC*, etc.) and 6 down-regulated ones (*NLRP1, GNAI1, CCNG1*, etc.) (Supplementary Table S8). It can be assumed that the knockdown of the *RPL29* gene impairs the balance of the p53-MDM2 system, which, in turn, triggers a chain of cellular events leading to changes in the activities of genes whose expression is regulated by p53. Another effect of the disruption of ribosome biogenesis is dysregulation of the c-Myc signaling pathway [40,41]. Although we did not find this pathway among those that could be affected by the eL29 deficiency and did not detect the *MYC* gene among DEGs, a comparison of the set of DEGs (Supplementary Table S4) with a group of c-Myc-regulated genes [38] allowed us to reveal 57 up-regulated and 55 down-regulated DEGs, which are c-Myc target genes (Supplementary Table S9). The reorganization of the expression of these genes in eL29-reduced cells may be caused by disruption of the interaction of c-Myc with some of its partners, similar to that proposed above for p53-regulated ones. In general, the appearance of p53 and c-Myc target genes in the set of DEGs from eL29-knocked down cells indicates that an imbalance of eL29 in cells may result in the activation of processes associated with oncogenesis. This assumption is consistent with the modern concept regarding the impact of dysregulation of ribosome biogenesis and translation on the development of malignant cell transformation, although a complete understanding of the molecular basis of the intermediate processes leading to the above event has not yet been achieved [5,42].

## 4. Discussion

Thus, the analysis of RNA-seq data from HEK293 cells knocked down of the eukaryote-specific ribosomal protein eL29, which is considered a non-essential component of the translational machinery, revealed a significant restructuring of the transcriptome profile caused by eL29 deficiency. Our findings show that an approximately 2.5-fold reduction in the eL29 level does not affect cell viability, but leads to considerable changes in the expression of 1308 genes at the transcription level, which are manifested in an increase or decrease in their activities, thereby reflecting a reorganization of regulation of gene expression in response to eL29 deficiency. Although the data obtained with immortalized eL29-knocked down HEK293 cells may not fully correspond to the effects of eL29 deficiency in specialized cells of organisms, nevertheless, the information gained indicates the importance of this protein for maintaining the balance of gene products implicated in certain cell pathways.

Although the eL29 insufficiency led to a suppression of the expression of genes associated with rRNA processing and ribosome biogenesis, cellular mRNA contents for most ribosomal proteins were not changed significantly. At the same time, the mRNA levels of the proteins uL5, uL11, uL18, eL22, and eL34, components of the 60S ribosomal subunit, were reduced, which indicates that the expression of the genes encoding these proteins at the transcription level is not synchronized with the expression of other ribosomal protein genes. Perhaps the genes of these five proteins are controlled by specific mechanisms that are different from those regulating the cellular balance of the remaining ribosomal proteins, and the knockdown of eL29 may somehow affect these ones. For instance, as mentioned in the Introduction, uL11, which, like eL29, is considered a non-essential ribosomal protein [10], is able to control its own synthesis through inhibiting the splicing of its pre-mRNA [15], that is, a mechanism different from that based on MDM-mediated degradation by which cells maintain the balance of many ribosomal proteins [39,40]. Besides, the proteins uL5 and uL18 are assembled into the 60S ribosomal subunit when they are already bound to 5S rRNA that is synthesized by RNA polymerase III, unlike other rRNAs synthesized by RNA polymerase I [43]. This may suggest that the syntheses of these proteins and 5S rRNA in cells are regulated in a coordinated manner, regardless of the balance of all other ribosomal proteins.

Notably, eL29 deficiency was found to cause changes in the expression of a number of genes that are targets for p53 or c-Myc, without affecting the activity of the *TP53* and *MYC* ones themselves. Among these genes, there are *BAX* encoding BCL2-associated X protein that is a regulator of apoptosis, *MKNK2* encoding MAPK-interacting serine/threonine kinase 2 that plays an important role in oncogenic cell transformation and malignant cell proliferation, as well as *PIDD1* encoding p53-induced death domain protein 1 that is an effector of p53-dependent apoptosis and a number of others. All this strongly hints that the imbalance of eL29 may be associated with oncogenesis.

The obtained data on the viability of eL29-reduced HEK293 cells are consistent with the observation that mice with homozygous eL29-null mutation are able to survive despite various growth abnormalities [27,28]. Although it is difficult to judge the causes of these specific disorders, the appearance of a phenotype with reduced skeleton size and bone fragility in such mice could be related to a significant dysregulation of the *COL24A1* gene (LFC = 1.00, Supplementary Table S4) encoding fibrillar collagen, which is expressed in bones and regulates type I collagen fibrillogenesis [44,45], due to the lack of eL29. In addition, our findings explain the fact reported in the aforementioned studies that the sizes of the body and organs in eL29-knocked out mice are also proportionally reduced. As noted in the Introduction, the authors have believed that the cause of these defects was ribosome insufficiency resulting from the lack of eL29. Our data showed that, although eL29-null ribosomes can participate in translation, a reduction in the level of this protein in cells leads to a decrease in the activities of genes associated with processes that ensure the biogenesis of ribosomes and control their functioning during translation. Consequently, growth defects in mice lacking eL29 have most likely been mediated by a deficiency of products of genes associated with the above processes, resulting from a decrease in their expression levels, which has led to the insufficiency of ribosomes and the dysregulation of translation. According to our data, these genes are those encoding DExD-box helicases 21 and 52, WD repeat-containing proteins 12, 36 and 43, RIO kinase 3, UTP3 small subunit processome component, pre-rRNA-processing protein TSR1, as well as eukaryotic translation initiation factor 2 alpha kinase 2, eukaryotic translation initiation factor 4 gamma 2 and other proteins.

On the other hand, a lack of ribosomes and the violation of translational control can lead to a deficiency of some specific proteins and thereby trigger mechanisms that activate the expression of the corresponding genes. Therefore, it is rational to suggest that the enhanced expression of certain genes in eL29-reduced HEK293 cells is a consequence of the deficiency of the products of these genes caused by the above reasons. Such genes are those that encode G3BP stress granule assembly factor 2 (G3BP2), mitochondrial RNA polymerase (MtRPOL), DExH-box helicase 34 (DHX34), nucleolar and coiled-body phosphoprotein 1 (NOLC1), activator of 90 kDa heat shock protein ATPase homolog 1 (AHA1), mitochondrial protein adenylyltransferase SelO (SelO), tyrosine-protein kinase CSK (CSK) and many others.

Finally, since it is known that eL29 as a heparin/heparan sulfate binding protein is involved in cell-to-cell communication and adhesion [22], it is not surprising that a decrease in its cellular level causes activation of a number of genes associated with pathways related to collagen formation and Golgi vesicle transport. Among these genes, there are those encoding chains of numerous collagens (collagen type IV alpha 1, 2 and 6 chains, as well as collagen types VI, XIII and XXIV alpha 1 chains), insulin-like growth factor 2 receptor, transmembrane P24 trafficking protein 9, dynactin subunit 1 and others.

## 5. Conclusions

Determining the effect of the deficiency of the ribosomal protein eL29 in mammalian cells on the transcriptome profile made it possible to establish a relationship between the cellular balance of eL29 and the activities of certain genes and to reveal processes in which proteins encoded by eL29-dependent genes are involved. Among the up-regulated genes, there are many implicated in cell responses to improperly folded proteins, activation of chaperones, stress of the endoplasmic reticulum, as well as in the regulation of GTPase activity and intracellular signal transmission. The down-regulated genes are preferably related to ribosome biogenesis, nuclear transport, translation regulation, and antiviral mechanisms. The occurrence of targets for p53 and c-Myc among eL29-dependent genes may indicate a link between a decrease in the level of eL29 in cells and their malignant transformation. Identifying the pathways through which eL29 contributes to the cellular mechanisms that control the above events could be the next frontier of research in this area.

## Figures and Tables

**Table 1:** GO enrichment analysis data for DEGs found with eL29-knocked down HEK293 cells ^1^.

GO Term	Definition	Fold Enrichment
	**For up-regulated DEGs:**	
GO:0006515	protein quality control for misfolded or incompletely synthesized proteins	6.54
GO:0034976	response to endoplasmic reticulum stress	2.76
GO:0007005	mitochondrion organization	2.39
GO:0043087	regulation of GTPase activity	2.09
GO:0007346	regulation of mitotic cell cycle	2.09
	**For down-regulated DEGs:**	
GO:0006413	translational initiation	3.37
GO:0022613	ribonucleoprotein complex biogenesis	2.87
GO:0008654	phospholipid biosynthetic process	2.70
GO:0051169	nuclear transport	2.61
GO:0090407	organophosphate biosynthetic process	2.52
GO:0006417	regulation of translation	2.48
GO:0031346	positive regulation of cell projection organization	2.35
GO:0072657	protein localization to membrane	2.17
GO:0016032	viral process	2.11
GO:0006396	RNA processing	2.08
GO:0055086	nucleobase-containing small molecule metabolic process	2.08
GO:0031329	regulation of cellular catabolic process	2.07
GO:0007015	actin filament-based process	2.03
GO:0080135	regulation of cellular response to stress	2.01

^1^ The GO terms with fold enrichment values larger than 2, which are the highest in the hierarchical tree, are presented.

## Supplementary Materials

The following are available online at https://www.mdpi.com/2073-4409/9/5/1228/s1, Figure S1: Key quality control metrics for raw and mapped RNA-seq data, Figure S2: RT-qPCR analysis of gene expression in eL29-knocked down HEK293 cells, Figure S3: Gene expression of ribosomal proteins in eL29-knocked down HEK293 cells compared to that in those transfected with scrambled siRNA, Table S1: Sequences of siRNAs used, Table S2: Basic characteristics of the obtained cDNA libraries, Table S3: Sequences of oligonucleotides used for RT-qPCR, Table S4: A gene set obtained from RNA-seq data using DESeq2, which was used for the analysis of differential expression, Table S5: A set of genes with statistically significant changes in their expression (designated as DEGs) in eL29-knocked down HEK293 cells, Table S6: The DEGs group encoding ribosomal proteins, Table S7: A list of DEGs-related processes according to pathway analysis, Table S8: A set of DEGs overlapping with that of 343 p53-target genes described in [5], Table S9: A set of DEGs overlapping with that of 1469 c-Myc-target genes described in [6].
