# Supplementary material for: Knockdown of the Ribosomal Protein eL29 in Mammalian Cells Leads to Significant Changes in Gene Expression at the Transcription Level
Source: Cells. 2020 May 15;9(5):1228. doi: 10.3390/cells9051228 (PMC7291024; doi:10.3390/cells9051228)
Supplement: Supplementary file 1 [file cells-09-01228-s001.zip › Supplementary figures and tables.pdf]

Supplementary figures and tables.

Figures

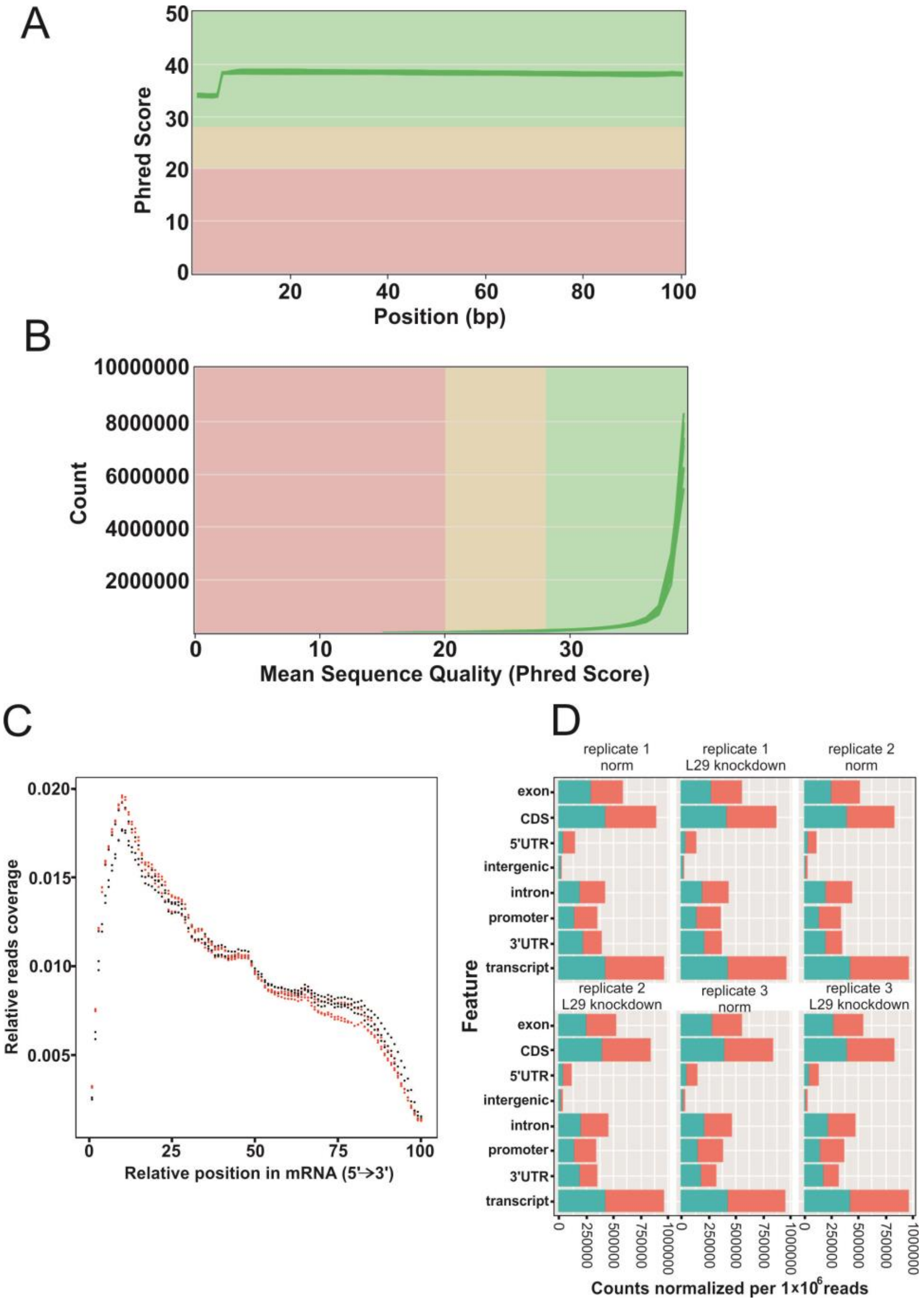

Figure S1. Key quality control metrics for raw and mapped RNA-seq data. (A) The FastQC Mean Quality Scores plot for all replicates generated by the MultiQC tool, which shows the mean quality values across each nucleotide base position in the read. The red, yellow and green areas of the plot correspond to Phred Score values < 20, between 20 and 28, and > 28, respectively. (B) The FastQC Per Sequence Quality Scores plot for all replicates generated by the MultiQC tool, which displays the number of reads with average quality scores. The color areas as in A. (C) Relative reads coverage of the 1000 most abundant transcripts (according RPKM values). Black and red dashed lines correspond to replicates obtained from HEK293 cells

transfected with scrambled siRNA and from eL29-knocked down ones, respectively. (D) The plot of the distribution of reads across annotation features for any read length generated by the systemPipeR. The red and blue parts of the columns correspond to the sense and antisense strands of the reads, respectively.

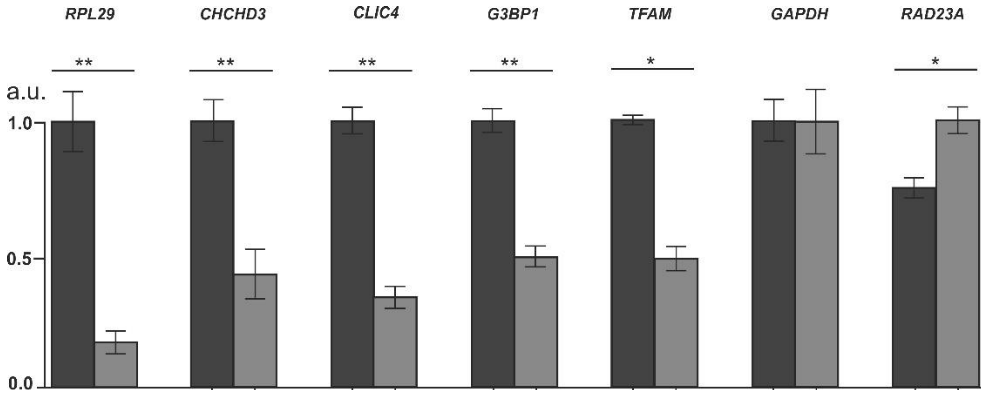

Figure S2. RT-qPCR analysis of gene expression in eL29-knocked down HEK293 cells. The relative contents of mRNAs for several genes (designated at the top) in cells transfected with scrambled siRNA or eL29 mRNA-specific siRNAs are presented in arbitrary units (a.u.) as black and grey columns respectively. Error bars are S.D. from three biological replicates; *P < 0.05, **P < 0.01, Mann-Whitney test.

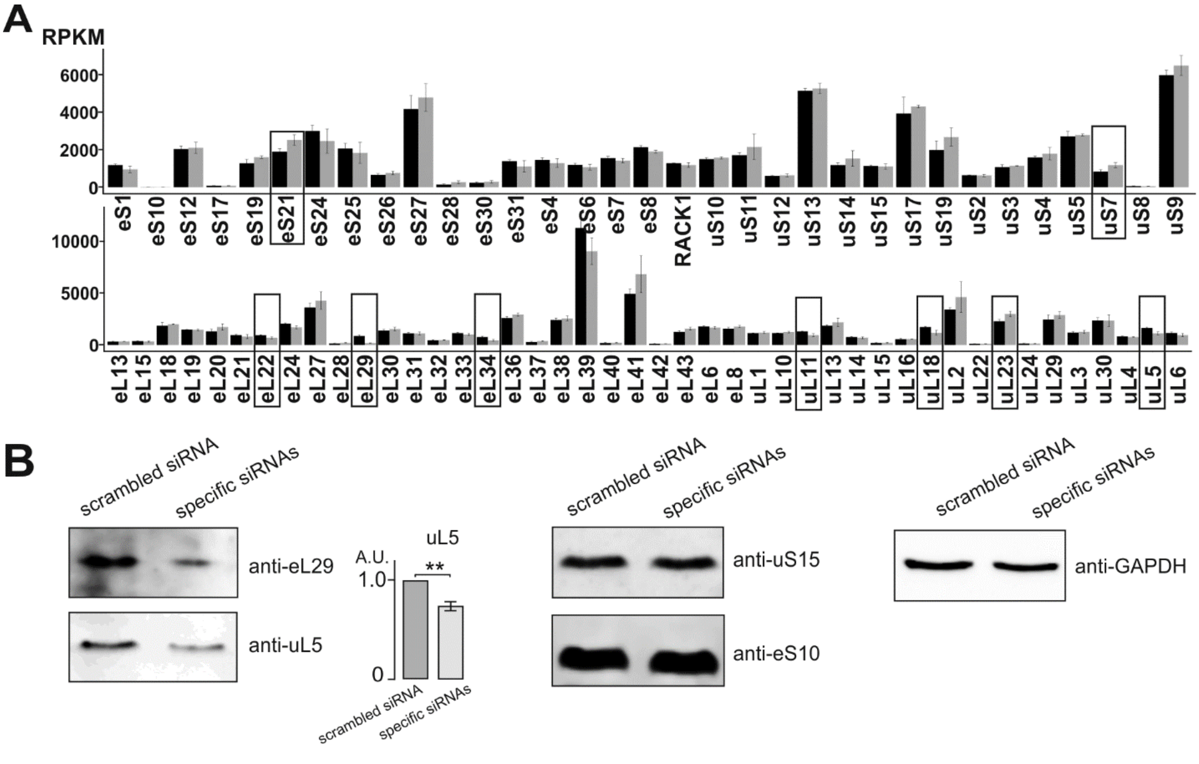

Figure S3. Gene expression of ribosomal proteins in eL29-knocked down HEK293 cells compared to that in those transfected with scrambled siRNA (taken as normal cells). (A) The

RPKM values bar plots for genes encoding proteins of 40S (upper panel) and 60S (bottom panel) ribosomal subunits in normal cells (black columns) and eL29-knocked down cells (grey columns) generated for 3 biological replicates. The error bars shows the standard deviation. (**B)** The western blot analysis of the contents of eL29 and uL5, as well as uS15, eS10 and GAPDH (taken as controls) in HEK293 cells transfected with scrambled siRNA or eL29-specific siRNAs. Diagram shows the data of the western blot analysis for uL5 obtained in triplicate as the mean of arbitrary units (A.U.) ± CEM (**p < 0.01, calculated using Student's t-test).

Table S1. Sequences of siRNAs used.

**Duplex number and oligonucleotide name**	**Sequence**	**Source**
I. L29 mRNA-specific-sense	5'-gcgugcucgugcccguauu**dTdT**-3'	[1,2]
I. L29 mRNA-specific-antisense	5'-**phosphate**-aauacgggcacgagcacgc**dTdT**-3'	[1,2]
II. L29 mRNA-specific-sense	5'-ggccaaggccaaggaucaa**dTdT**-3'	[1]
II. L29 mRNA-specific-antisense	5'- **phosphate**-uugauccuuggccuuggcc**dTdT**-3'	[1]
III. Scrambled-sense	5'-uucuccgaacgugucacgu**dTdT**-3'	[1,3,4]
III. Scrambled-antisense	5'- **phosphate**-acgugacacguucggagaa**dTdT**-3'	[1,3,4]

Table S2. Basic characteristics of the obtained cDNA libraries.

**Replicate, batch, code**	**Definition**	**Platform**	**Raw reads count**	**Read type**	**Total reads mapped to genome (%)**
Rep1, batch 1, 5-T07	Control with scrambled siRNA	HiSeq 2500	2x10110203	2x100 bp	19442986 (96.2)
Rep1, batch 1, 5-T08	eL29 knockdown	HiSeq 2500	2x12050076	2x100 bp	23232708 (96.4)
Rep2, batch 2, 8-T01	Control with scrambled siRNA	HiSeq 2500	2x12446417	2x100 bp	23834427 (95.7)
Rep2, batch 2, 8-T02	Control with scrambled siRNA	HiSeq 2500	2x12731297	2x100 bp	24350779 (95.6)
Rep3, batch 2, 8-T03	eL29 knockdown	HiSeq 2500	2x12818849	2x100 bp	24610696 (96.0)
Rep3, batch 2, 8-T04	eL29 knockdown	HiSeq 2500	2x13436686	2x100 bp	25794050 (96.0)

Table S3. Sequences of oligonucleotides used for RT-qPCR.

Gene	Forward primer (5'-3')	Reverse primer (5'-3')
RAD23A	gtgaaggtgctaaaggagaag	gggaaggatgtagaggact
TFAM	cgctccccttcagttttgt	acgagtttcgtcctctttagca
CHCHD3	ccaaagagctggaccgagag	agcatttgagggtctggtgg
G3BP1	caccacaaagacctcagcgg	ccccttcccactccaaatc
CLIC4	aatgaagcactggagagggg	tcactgggacaggtattggt
RPL29	cgacttgcctacattgcc	cctgagctggaactgaag
GAPDH	gtgaaccatgagaagtatgacaac	catgagtccttccacgatacc

Supplementary tables as Excel files.

Table S4. A gene set obtained from RNA-seq data using DESeq2, which was used for the analysis of differential expression.

Table S5. A set of genes with statistically significant changes in their expression (designated as DEGs) in eL29-knocked down HEK293 cells.

Table S6. The DEGs group encoding ribosomal proteins.

Table S7. A list of DEGs-related processes according to pathway analysis.

Table S8. A set of DEGs overlapping with that of 343 p53-target genes described in [5].

Table S9. A set of DEGs overlapping with that of 1469 c-Myc-target genes described in [6].

References:

1. Liu JJ, Huang BH, Zhang J, Carson DD, Hooi SC. Repression of HIP/RPL29 expression induces differentiation in colon cancer cells. *J. Cell. Physiol.* 2006, **207**, 287-292.
2. Li C, Ge M, Yin Y, Luo M, Chen D. Silencing expression of ribosomal protein L26 and L29 by RNA interfering inhibits proliferation of human pancreatic cancer PANC-1 cells. *Mol. Cell. Biochem.* 2012, **370**, 127-139.
3. Kim J, Ahn S, Ren XR, Whalen EJ, Reiter E, Wei H, *et al.* Functional antagonism of different G protein-coupled receptor kinases for beta-arrestin-mediated angiotensin II receptor signaling. *Proc. Natl. Acad. Sci. U. S. A.* 2005, **102**, 1442-1447.
4. Yoon JH, De S, Srikantan S, Abdelmohsen K, Grammatikakis I, Kim J, *et al.* PAR-CLIP analysis uncovers AUF1 impact on target RNA fate and genome integrity. *Nat. Commun.* 2014, **5,** 5248.
5. Fischer M. Census and evaluation of p53 target genes. *Oncogene* 2017, **36**, 3943-3956.
6. Kim J., Lee JH., Iyer VR. Global identification of Myc target genes reveals its direct role in mitochondrial biogenesis and its E-box usage in vivo. *PloS One* 2008, **3**, e1798.
